# Challenges in the standardization of innate immune status assessment

**DOI:** 10.3389/fimmu.2026.1753841

**Published:** 2026-03-16

**Authors:** Ying Liu, Tingfang Mao, Rong Chen, Hairong Luo, Shiyu Zhang, Feng Chen, Xiang Shen, Wan Li, Jinhui Liu, Guiping Guan, Zemeng Feng, Yulong Yin

**Affiliations:** 1 Hunan Provincial Key Laboratory of Animal Nutritional Physiology and Metabolic Process, National Engineering Laboratory for Pollution Control and Waste Utilization in Livestock and Poultry Production, Institute of Subtropical Agriculture, Chinese Academy of Sciences, Changsha, Hunan, China; 2 College of Veterinary Medicine, Hunan Agricultural University, Changsha, Hunan, China; 3 College of Bioscience and Biotechnology, Hunan Agricultural University, Changsha, Hunan, China; 4 College of Advanced Agricultural Sciences, University of Chinese Academy of Sciences, Beijing, China

**Keywords:** immune detection, immune status assessment, immunological evaluation technology, innate immunity, standardization

## Abstract

The initial defense against pathogens is the innate immunity, which is characterized by rapidity and the broad-spectrum operation that facilitates the rapid identification and removal of the invasion by microorganisms. The determination of innate immune status is a methodological approach to assessing the strength of innate immune defense and has been extensively utilized in vaccine development, disease surveillance, clinical stratification, and postoperative recovery. Nevertheless, discrepancies in indicator purposes, methodologies, and references are currently seen where quantitative judgments cannot rely on and be consistent. Hence, methodological innovation and analysis technologies need to be developed, and a standardized approach and common criteria should be created to address the increasing clinical and research needs of quantitative assessment. In the framework of human health and clinical practice, the given work gives a concise review of the key inadequacies of current approaches to innate immune evaluation, incorporates the modifications into the current assessment criteria, explains the drawbacks of modern technologies, and suggests an evaluation scheme involving many parameters to facilitate the standardization and quantification of the assessment of the innate immune status.

## 1 Introduction

**Figure 1:**
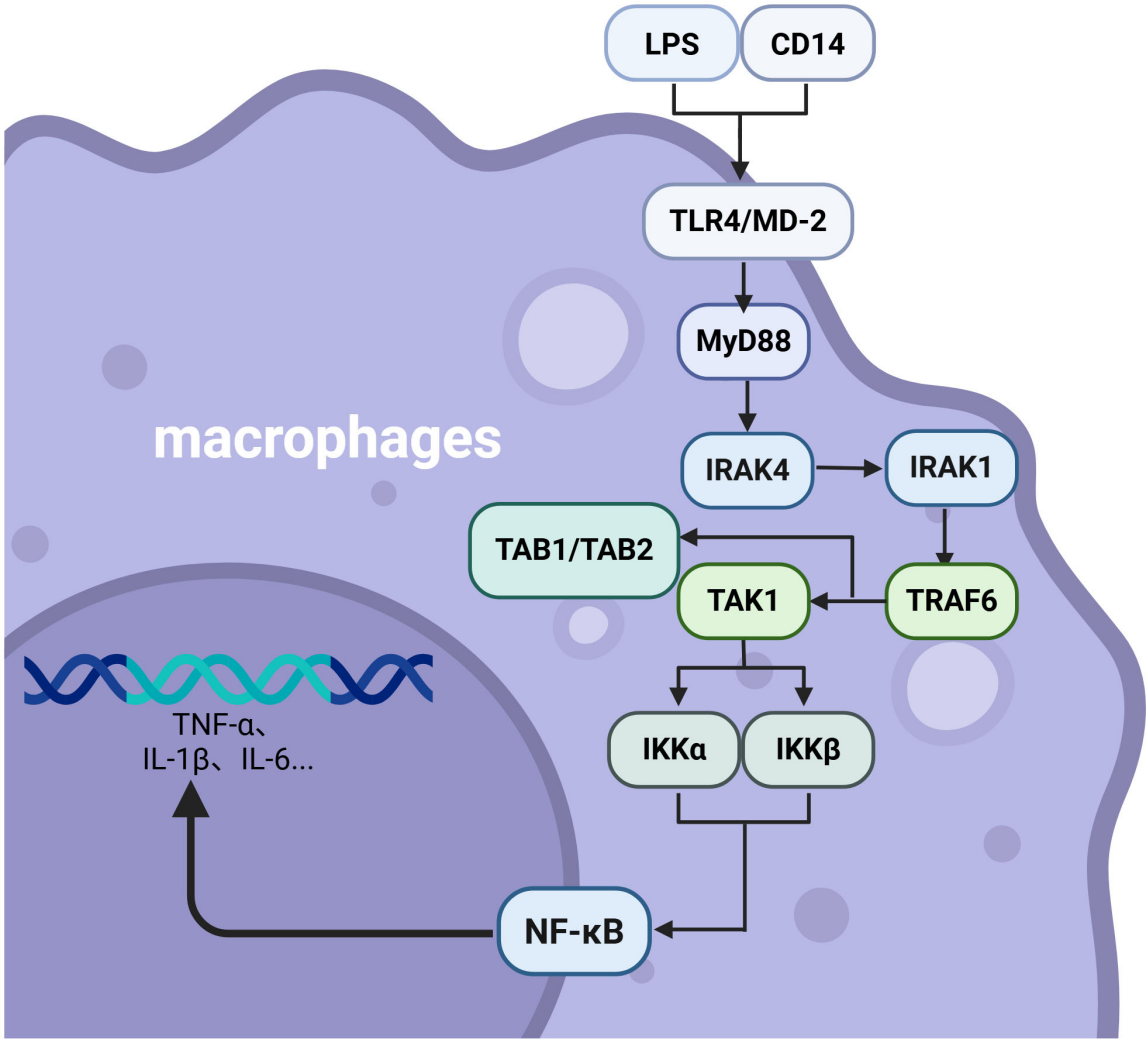
Schematic representation of LPS-triggered TLR4/MyD88-dependent signaling cascade in macrophages, leading to NF-κB activation and cytokine production.

The immune system is a major component of the body in terms of protection of health and the first line against ailments, as it is the main defense available to the body against pathogenic invasion. The presence of the natural immune response (innate immunity) is one of the primary immune response elements, and it is rapid with a lack of specificity (1). Such a defense system includes physical and chemical mechanisms, including skin and mucosal disposition, and internal immune resources, including immune cells, pattern recognition receptors (PRRs), and the complement system (2). The innate immune system is immediately triggered at the initial phase of pathogen invasion. It detects pathogen-associated molecular patterns (PAMPs) or damage-associated molecular patterns (DAMPs) released into the extracellular environment by injured tissues through PRRs, including Toll-like receptors (TLRs) and, in doing so, triggers inflammation, complement activation, and the production of cytokines. These mechanisms stimulate the signaling of alarm intra-cellularly and attract the effector cells, including the macrophages and natural killer (NK) cells (3–5). TLRs activate the NF-KB and MAPK products to mediate the production of pro-inflammatory cytokines, as illustrated in **Figure 1** (6).

**Figure 2:**
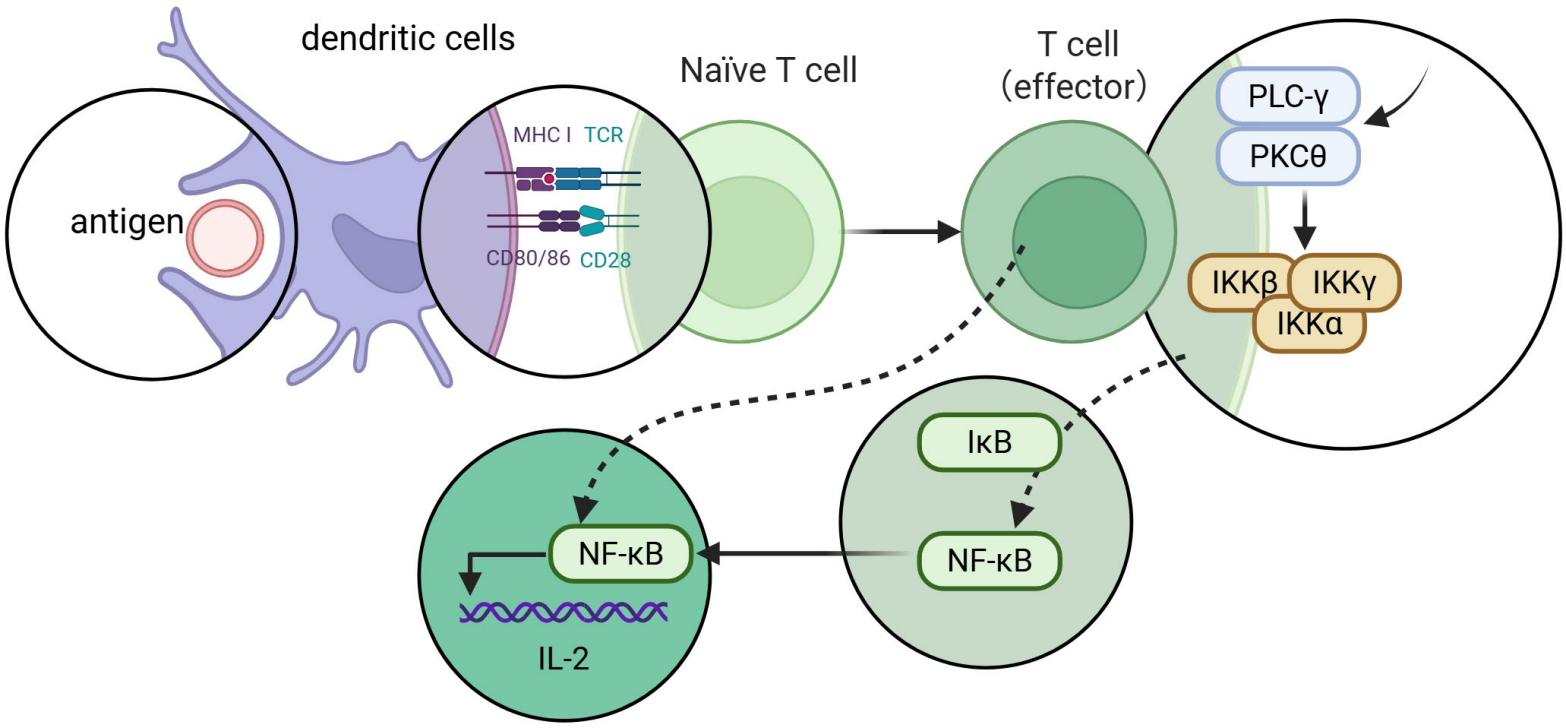
DCs activate naïve T lymphocytes through antigen presentation and co-stimulatory signaling, thereby triggering the NF-κB signaling pathway and promoting the expression of effector T-cell cytokines such as IL-2.

**Figure 3:**
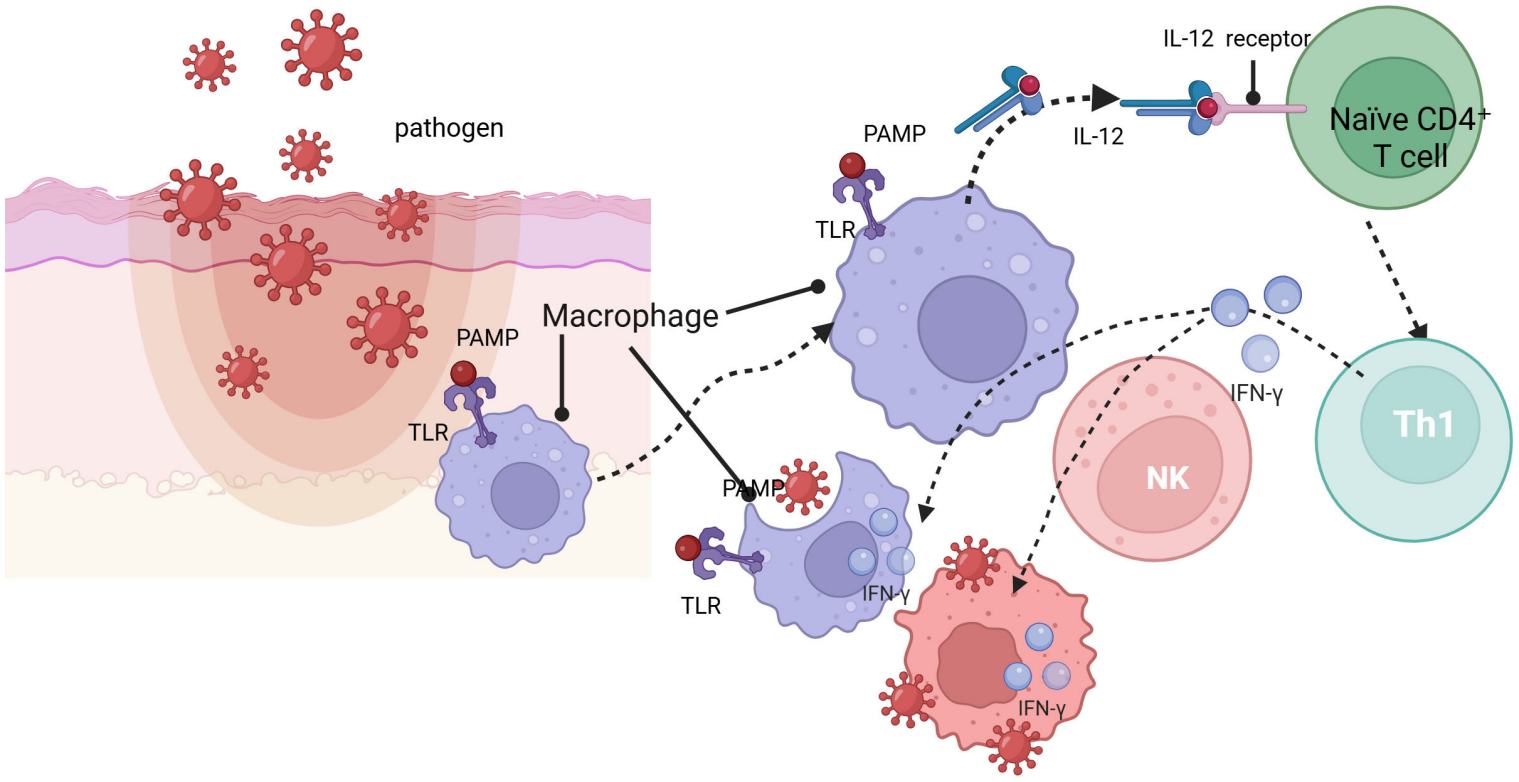
Upon pathogen invasion, DCs and macrophages recognize PAMPs via TLRs, leading to NF-κB activation. This subsequently induces the production of the pro-inflammatory cytokine IL-12, which promotes the differentiation of naïve CD4^+^ T cells into Th1 cells and stimulates the secretion of IFN-γ, further activating macrophages and enhancing their phagocytic and antigen-presenting capacities.

The direct protection offered by innate immunity creates the provisional protective context, which leads to the following stimulation of adaptive immune functions (7). Adaptive immunity is characterized by long-term protection and immunological memory, without which the first line of defense during the initial period of an infection is taken by innate immunity. Dendritic cells (DCs), NK cells, and cytokines are the mediators between the two arms of immunity and are intricately related, as well (8, 9). DCs and macrophages are crucial in the inception of the adaptive immunity through the ingestion of pathogens or through displaying the antigens to naïve T lymphocytes, as depicted in **Figure 2** (3, 10, 11). As shown in **Figures 3**, NK cells boost adaptive immunity through the destruction of infected cells and the control of inflammatory pathways.

**Figure 4:**
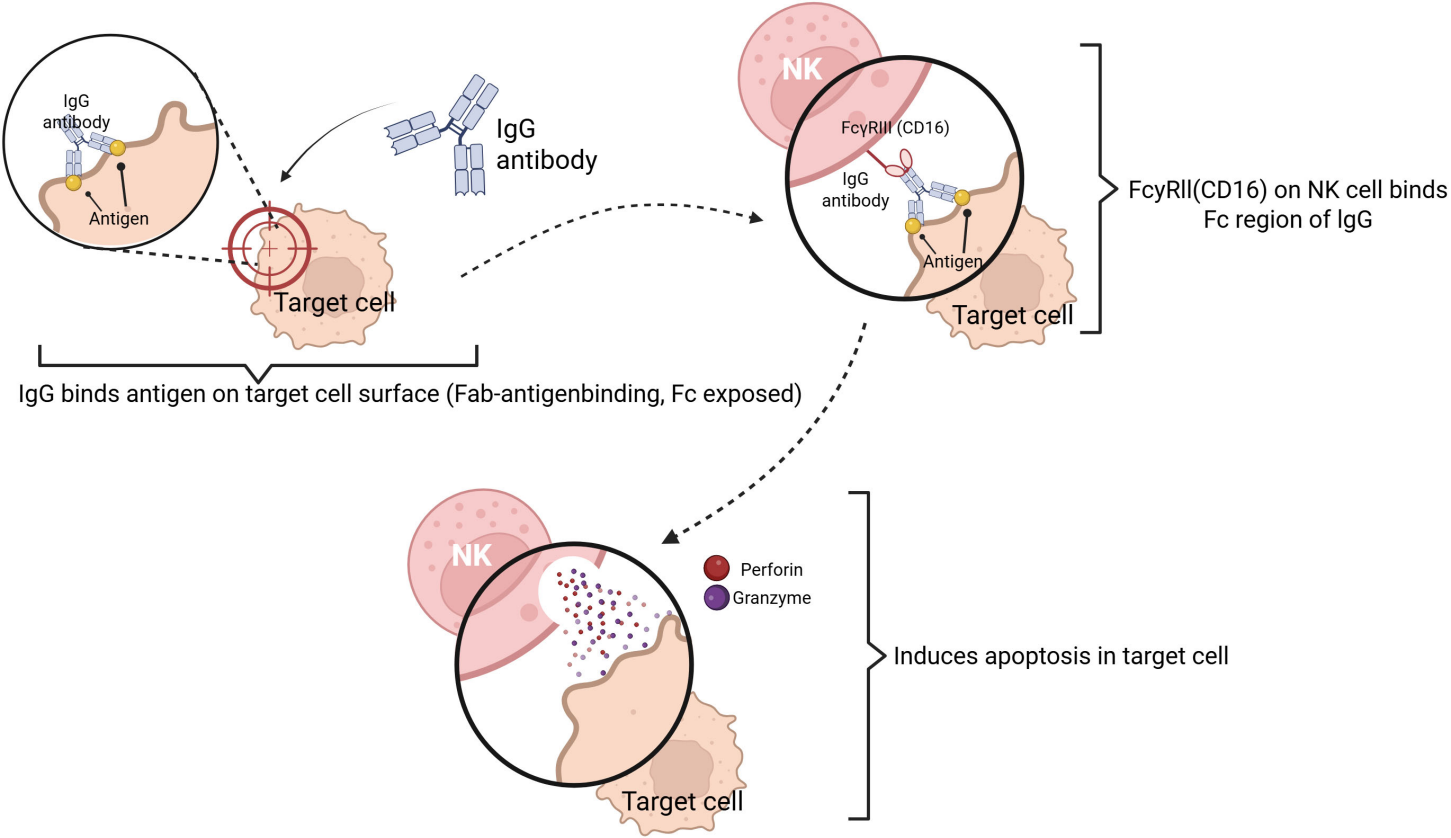
Antibody-dependent cellular cytotoxicity (ADCC) is a mechanism by which effector cells, such as NK cells, recognize and eliminate antibody-coated target cells through Fc receptor engagement.

Additionally, as **Figures 4** depicts, released cytokines in case of the activation of the innate immune system, like IL-12 and IFN-γ, also enhance T lymphocyte responses (12). Conventionally, innate immunity has been thought of as being able to augment future response with non-specific memory-like reprogramming of innate immune cells. However, recent research has found that innate immunity shows a type of immunological memory, which is the trained immunity (13, 14). Imbued with high conservation through the evolution of various species, innate immunity is a central part of the infection protection system and inflammatory responses, as well as the links closely with chronic inflammation, an imbalanced state, and recovery after a surgical intervention. Innate immune parameters in comparison with the complex adaptive immune system are typically less complex and more quantifiable and have a higher inherent level of transnationalism in basic science and medicine.

## 2 Fields and practical applications

The most widespread instance of assessing innate immune function in contemporary research is biomarker-based detection. As human health needs and clinical medicine have escalated, precise identification and detailed study of innate immune biomarkers have slowly shifted from being a lab-based study into clinical practice. The tests have significant purposes in the optimization of vaccines in their adjuvant, pre-operational, and chronic illness immune surveillance, in individual nutritional intervention, and geriatric healthcare management. The types of biomarker detection, such as ELISA and flow cytometry, are widely used to define the immune status, forecast the occurrence of the disease, and inform the clinical management approaches (1, 15–17). Another application of innate immune function assessment in biomedical research is the ex-planation of disease mechanisms and the control of the disease to generate an important diagnostic and monitoring method in the clinical management of tumors, autoimmune diseases, and other serious illnesses (18–20).

An in-depth knowledge of innate immune response is necessary in the rational design and optimization of next-generation vaccines in the context of vaccine development (21, 22). As an example, successful adjuvant strategies are capable of significantly boosting humoral immunity (23), whereas cross-species comparatively studies (such as in mice and non-human primates) can be of great use in designing also broad-spectrum protective vaccines. Innate immune responses studies have shown that there are major immune evasion mechanisms that happen during HIV infection and aid in the vaccine design (24). Likewise, the studies conducted on coronavirus vaccines have indicated that the increased arousal of innate immune responses raises the level of effectiveness of vaccines in general (25, 26). In immunodeficiency studies, the observation of innate immune biomarkers will help to diagnose them at an early stage and develop a personalized treatment plan (27). Studies of immune-deficiency disorders in infants born with such disorders, including severe combined immunodeficiency (SCID), show that the profile of cytokines and develop-mental monitoring of immune cells can give important directions that can be considered in a clinical decision (28). Moreover, early genetic screening has facilitated the detection of any latent cases of immune defects, which can be treated in time (29, 30). Innate immunity is the basis of preparing anti-infective means in the context of the necessary protection against bacterial, viral, and fungal infection acquired by humans, as it is a core of host defense (2, 31). Indicatively, innate immune cells like NK cells have been noted to be involved early on in studies conducted on influenza infection (32). Dysregulation of innate immunity is known to constitute the pathogenesis of diseases in autoimmune and allergic disease and cancer. Therefore, the active monitoring of certain cytokines or subsets of innate immune cells and the targeted correction of their upbringing have become one of the primary areas of investigation (33, 34). The study of the innate immune system supplies more evidence on how the host defenses are carried out and creates an academic basis for the development of new methods of action in medicine. The evaluation of innate immune functioning provides valuable advice on the accuracy of intervention and the development of evidence-based solutions in this critical reference to disease surveillance, clinical diagnosis, and health management.

## 3 Limitations in innate immune status assessment

There are also notable challenges in current approaches to assessing innate immunity, such as no stringent selective criteria of measurement indicators, experimental techniques innovation requirement, and no uniform assessment system to be used in operational protocols and results interpretation. A proper systematic picture, which can completely explain the innate immune condition, has not yet been realized.

### 3.1 Indicator selection and multi-parameter analysis

Different biomarkers are now regarded as essential clues to the evaluation of immune functioning with the development of innate immune monitoring technology. Among them are cytokines, PRRs, and acute-phase proteins like C-reactive protein (CRP) (35, 36). Immune indicators indicate the status of the innate immune system and occur at molecular, cellular, and systemic levels. The indicators of immune activity on the molecular level are AMPs (e.g., lysozyme) and inflammatory mediators (e.g., IL-1β) (37), and the dendritic cell antigen presenting capacity is often scrutinized through an upsurge of MHC-II on the surface during maturation (22). Neutrophil chemotax is and phagocytic activity can be used as a reference parameter at the cellular level (38). Systemic immune activity is commonly determined based on serum CRP and pathogen challenge tests, which offer the direct result of host defense to the external pathogens (35, 36). The intricate biological mechanisms of innate immunity are being uncovered slowly by modern molecular and cellular methods. Nonetheless, coherent indicator systems and testing procedures cannot be readily included in various experimental modes. To be more precise, multi-dimensional immune measurements necessitate the incorporation of ratio measurements (e.g., neutrophil-to-lymphocyte ratio, NLR), composite scoring, or dynamic trend assessments to enhance the meaning-making process. As an example, the integrated use of NLR and CRP has already been used to predict the early subclinical infection of animals (39).

#### 3.1.1 Complexity of indicator selection

Signaling pathways are highly interdependent and dynamically controlled to mediate innate immune functioning (2, 40). The determination of the activity of these pathways and the choice of the best informative and com-prehensive markers, while maintaining the quality of the analysis and methods, is a significant challenge (41). PRRs mediate core mechanisms of innate immunity and, depending on a variety of pathways, including NF-κB and MAPK, regulate the production of cytokines and the activation of immune cells (42, 43). TLR promotion is capable of inducing different immune responses. As an example, in most cells, the TLR4 signaling catalyzes inflammation through the NF-κB pathway; however, the end results are cell type-specific: in macro-phage cells, TLR4 stimulates pro-inflammatory response, whereas in DCs it can trigger immune response or tolerance depending on the context (44, 45).

#### 3.1.2 Indicator ranges

The dynamic levels of immune markers and clinical context are very important in the interpretation of immune markers. As an illustration, high CRP is a common indication of acute infection, whereas continuous low-grade high increases can represent chronic inflammatory response, immune senescence, or high vulnerability to autoimmune disease (46). Equally, when healthy people have their baseline levels of IL-6 measured, the values are extremely low (<10 pg/mL), although an acute infection may cause a more than 100-fold rise (47). The transient elevation of IL-6 is one of the normal indications of the defense mechanism and functions as a defense against pathogen clearance, but once the levels become high, after pathogen clearance, IL-6 changes its defense properties into a disease-inducing agent, a contributor to chronic inflammation, immune dysregulation, and risk of developing dis-eases like diabetes and cardiovascular conditions (39, 48). Thus, the overall assessment of immune function needs to be found on the detailed analysis of numerous biomarkers and the development of ranges of values used to define the difference between physiological variability and pathological dysregulation (49, 50).

#### 3.1.3 Multi-parameter joint analysis

Instead of the absolute value of one single marker, the actual condition of the immune system is determined by dynamic hedges between pro- and anti-inflammatory forces and activation and suppression cues. It is important to know the correlations between the generally tested biomarkers of innate immunity to evaluate them comprehensively. Systems of multi-dimensional biomarkers should consider four categories, namely: inflammatory mediators, cellular function, metabolic status, and epigenetic control. Examining absolute changes will address issues with immune status interpretation. To illustrate, numerous pro-inflammatory cytokines (IL-1β, IL-6, TNF-α, IL-8) are interdependent in terms of their effects on infection and inflammation and work together with anti-inflammatory cytokines, i.e., IL-10, TGF-β (51). As far as inflammatory balance is concerned, TNF-α/IL-10 ratio is proven to be rele-vant to reflect the level of inflammatory imbalance in chronic liver disease, rheumatoid arthritis, and severe COVID-19 (52–55). An immune-metabolic coupling could be evaluated based on such indicators as the NAD +/NADH ratio (56). IL-8/IL-10 ratio demonstrates characteristic changes in sepsis in critical care, and the IL-1 cytokine can be indicative of the acute immune maladjustment (57–61). The rheumatoid arthritis measurement can be assessed better through joint analysis strategies, including measuring CRP and IL-6 simultaneously (62). There are significant negative correlations between certain indicators; for example, patients with higher serum IL-6 levels have a lower ability of their natural killer cells to express granzyme A (63). Proper analysis of the inherent immune activity involves a combination of various biomarkers, especially significant ratios, with case history.

### 3.2 Innate immune status assessment-related detect methods

**Figure 5:**
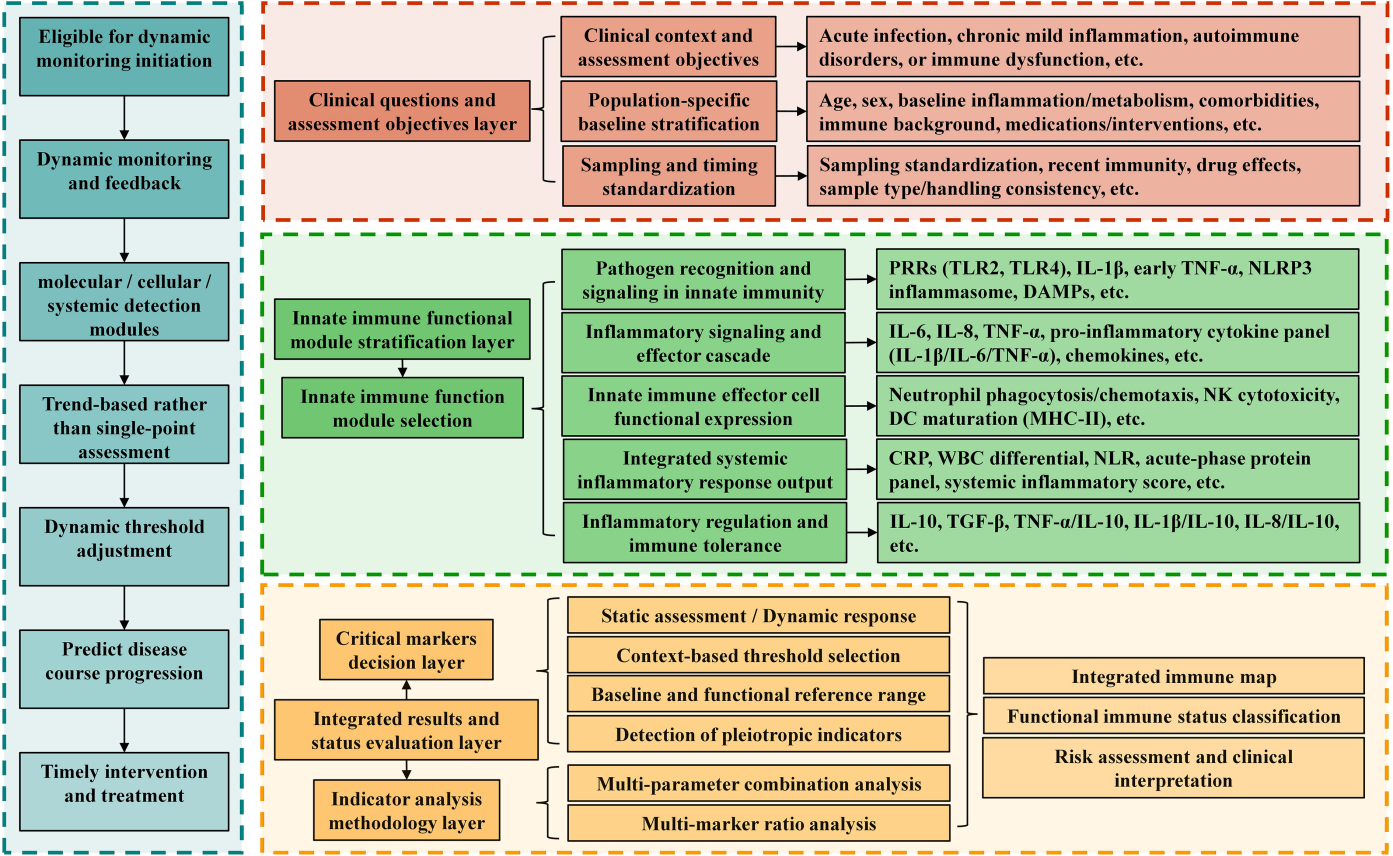
A multiparametric, function-oriented framework for innate immune assessment integrating clinical context, population stratification, sampling standardization, and innate immune functional modules. The framework emphasizes modular selection of innate immune functions, trend-based evaluation rather than single-point measurements, and dynamic threshold adjustment. By linking molecular, cellular, and systemic immune readouts with clinical decision layers, this approach supports comprehensive immune status classification, risk assessment, and individualized clinical interpretation.

**Figure 6:**
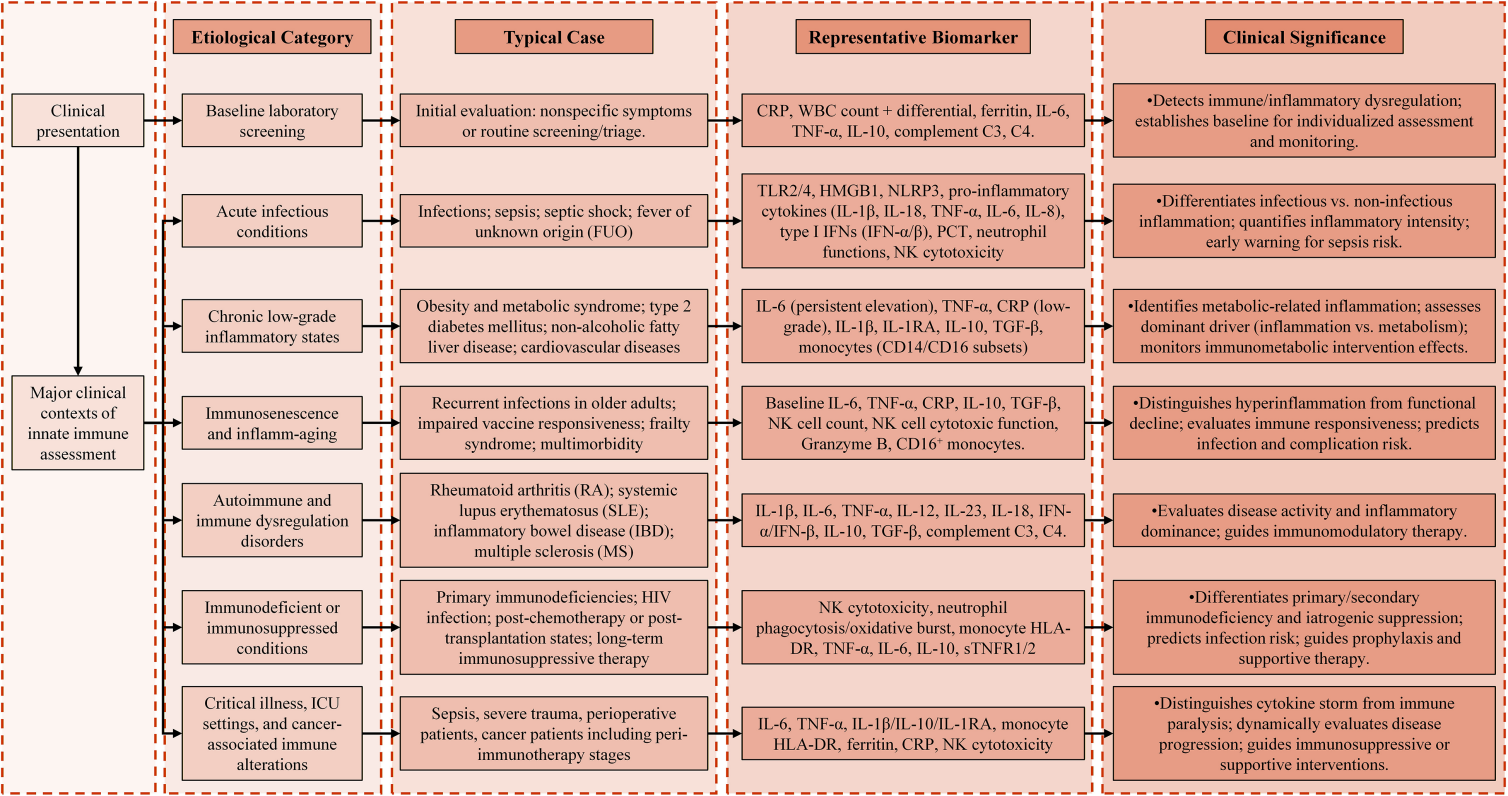
Schematic representation of clinical context–driven innate immune biomarker selection. Major clinical scenarios—including acute infection, chronic low-grade inflammation, immunosenescence, autoimmune disease, immunodeficiency, and critical illness—are linked to representative innate immune biomarkers and their clinical significance. The diagram highlights the complementary roles of functional, regulatory, and systemic inflammatory indicators in immune status evaluation, emphasizing integrated interpretation rather than reliance on single biomarkers.

Immunological techniques are now extensively employed in clinical settings for human health assessment, and **Table 1** outlines commonly used assays and the innate immune components they evaluate. The methods of immunological assessment are constantly being improved and have a higher level of sensitivity and specificity. However, there are still challenges in accurately identifying low-level immune disorders, especially in subclinical states or chronic inflammation-related diseases. Traditional immunological techniques (such as ELISA, flow cytometry, and PCR assays) remain fundamental to innate immune assessment due to their standardized workflows, high reproducibility, and well-established clinical interpretability. However, these methods suffer from insufficient sensitivity when target protein concentrations are low, limiting their utility in non-overt disease states (64). PCR and sequencing methods are sensitive to pathogen detection, but their common clinical use is constrained by a complex nature of operations and high costs (65). The in-consistency in the method of technique choice between studies and methodological standards and interpretation rules makes it difficult to integrate immunological datasets and restrict clinical translation. The typical methods of innate immune systems and their strengths and shortcomings have been summarized in **Table 2**. As shown in **Table 3**, emerging technologies such as digital immunoassays, multiplex detection platforms, and mass spectrometry enable the acquisition of highly sensitive and high-resolution innate immune biomarker profiles. These prove more suitable than traditional immunological techniques for complex tissue microenvironments (66). Nevertheless, these novel techniques still face challenges regarding assay stability, inter-platform reproducibility, data standardization, and clinical interpretability. To address these limitations, a multiparametric and function-oriented assessment framework is required. **Figures 5** illustrates an integrated, context-aware framework for innate immune function assessment, in which clinical objectives, population stratification, sampling standardization, and functional immune modules are jointly considered. Effective innate immune assessment relies on rational biomarker selection tailored to specific clinical contexts. **Figures 6** presents a structured diagram linking major clinical scenarios with representative innate immune biomarkers and their clinical significance. Whilst conventional methods retain irreplaceable advantages in operational standardization, result comparability, and long-term clinical validation, routine clinical practice similarly relies on complex algorithmic processing and advanced technical support for assay results. New technologies and traditional methods do not represent a simple substitution relationship, but rather than form complementary approaches across different detection targets, sample conditions, and application scenarios. Combinations of these biomarkers and analytical solutions have the potential to improve the knowledge of the innate immune system and serve as a scientific input to diagnose diseases, administer immunological treatment, and produce vaccines. It is highly necessary to devise coordinated, non-disciplinary assessment strategies and to design standardized systems of innate immunity assessment that consider factors of indicator selection, technical aspects, and clinical utilization, which will enhance the accuracy, reliability, and applicability of results. It is needed to establish a coordinated, interdisciplinary assessment framework that standardizes the procedures for selecting indicators, technical selection, quality control, and interpretation of results. It will enable the development of an assessment system for innate immunity that is scientifically sound, reproducible, and clinically applicable.

**Table 1:** Innate immune assessment methods and corresponding biomarkers.

Detection technique	Biomarker
Real-Time Quantitative PCR (qPCR)	IL-6, TNF-α, IFN-γ, IL-10, CRP, SAA, IgG, IgA, IgM, CD3, CD19, COX-2, iNOS, GSH, SOD, C3, C4, PCT, Fatty acids, Insulin resistance markers
Enzyme-Linked Immunosorbent Assay (ELISA)	IL-6, TNF-α, IFN-γ, IL-10, CRP, SAA, IgG, IgA, IgM, COX-2, iNOS, MDA, GSH, SOD, C3, C4, PCT, Fatty acids, Insulin resistance markers, Proteins in Tears, Urine, Lymph fluid
Western Blot	​IL-6, TNF-α, IFN-γ, IL-10, CRP, SAA, IgG, IgA, IgM, CD3, CD19, COX-2, iNOS, GSH, SOD, C3, C4, PCT, Insulin resistance markers, Proteins in Urine
Flow Cytometry	CD4+ T cells, CD8+ T cells, B cells, NK cells, Macrophages, Dendritic cells, Treg cells, CD3, CD19, Intracellular IFN-γ, Proteins in Lymph fluid
Immunohistochemistry/Immunofluorescence (IHC/IF)	IL-6, TNF-α, IFN-γ, IL-10, CRP, SAA, IgG, IgA, IgA, IgM, CD3, CD19, COX-2, iNOS, GSH, SOD, C3, C4, PCT, Proteins in Tears, Proteins in Lymph fluid
Mass Spectrometry	IL-6, TNF-α, IFN-γ, IL-10, CRP, SAA, IgG, IgA, IgM, CD3, CD19, COX-2, iNOS, MDA, GSH, SOD, C3, C4, PCT, Fatty acids, Insulin resistance markers, Proteins in Tears, Urine
*In Vitro* Functional Assays	Neutrophil phagocytosis, Neutrophil oxidative burst, NK cell cytotoxic activity

**Table 2:** Common techniques for innate immune status assessment.

Detection technique	Primary application	Advantages	Limitations	References
Real-Time Quantitative PCR (qPCR)	Precise quantification of mRNA expression levels of specific genes	High sensitivity and specificity; accurate quantification; relatively high throughput; cost-effective	Detects mRNA levels only, not functional protein abundance.	(75–77)
			Strongly dependent on primer efficiency and stable reference genes.	
			Potential amplification bias and nonspecific amplification.	
Enzyme-Linked Immunosorbent Assay (ELISA)	Quantitative detection of specific soluble proteins in fluids	High specificity and sensitivity; relatively simple operation; amenable to automation; suitable for clinical and large-scale screening	Detects only soluble proteins; unable to provide cellular localization.	(78–81)
			Typically measures one analyte per assay, limited multiplexing capacity.	
			Narrow dynamic range; less sensitive to extremely high or low concentrations.	
Western Blot	Detection of specific protein expression, molecular weight, and post-translational modifications	Provides information on protein molecular weight; validates antibody specificity; semi-quantitative	Semi-quantitative, with limited accuracy and reproducibility.	(82–84)
			Strongly dependent on antibody quality and specificity.	
			Low throughput; labor-intensive and time-consuming; limited quantitative precision.	
Flow Cytometry	Phenotypic analysis: identification, classification, and counting of immune cells.	Simultaneous analysis of multiple markers in a single experiment.	Technically complex; requires expert operation and data interpretation.	(68, 85–89)
	Functional analysis: detection of intracellular cytokines, phosphorylated signaling molecules, proliferation, apoptosis.	Single cell resolution provides insights into cellular heterogeneity.	High cost: antibody panels require optimization and validation.	
	Functional assays: evaluation of phagocytosis, ROS production, etc.	High throughput and robust statistical power.	Lacks spatial localization information within tissues.	
Immunohistochemistry/Immunofluorescence (IHC/IF)	Localization and visualization of protein expression and spatial distribution in tissue sections	Preserves tissue morphology and spatial context.	Semi-quantitative or qualitative; limited quantitative precision.	(90–93)
		Enables direct visualization of protein expression in specific cells or regions.	Interpretation may involve subjectivity.	
			Low throughput: antibody penetration and specificity remain challenges.	
*In Vitro* Functional Assays	Direct evaluation of immune cell functions such as macrophage/neutrophil phagocytosis and oxidative burst activity	Directly reflects cellular functionality; results are intuitive and highly correlated with physiological states	Experimental conditions critically affect results; strict controls required.	(94–96)
			Lack of methodological standardization, leading to reproducibility challenges.	
			Some probes may exhibit nonspecific reactivity.	

**Table 3:** Comparison of conventional and emerging technologies for innate immune assessment.

Dimension	Conventional immunological methods (e.g., ELISA, flow cytometry, PCR)	Emerging technologies (e.g., spatial omics, digital immunoassays, multiplex platforms)
Primary Targets	Single or limited soluble cytokines, surface markers, or nucleic acid sequences	Multiple immune biomarkers, cell subsets, and spatial cellular contexts
Analytical Sensitivity	Moderate; limited for low-abundance proteins or weak immune signals	High sensitivity, suitable for detecting low-grade immune dysregulation and subclinical states
Analytical Specificity	Dependent on antibody or primer quality; susceptible to cross-reactivity	Enhanced specificity through digital readout or spatial resolution strategies
Background Signal Interference	Relatively high in complex biological matrices	Effective background suppression and improved signal-to-noise ratio
Spatial Information	Generally unavailable; reflects bulk measurements	Provides cell- and tissue-level spatial localization of immune signals
Multiplexing Capacity	Limited; multi-marker analysis often requires sequential assays	High-throughput, simultaneous multi-parameter analysis
Sample Processing Complexity	Mature workflows with relatively simple handling procedures	Complex sample preparation and stringent quality requirements
Degree of Standardization	Well established protocols, reference ranges, and quality control frameworks	Incomplete standardization; limited cross-platform comparability
Data Interpretation	Intuitive and clinically interpretable	Highly dependent on computational analysis and bioinformatics pipelines
Reproducibility and Robustness	Extensively validated with good inter-laboratory reproducibility	Influenced by platform-specific algorithms and experimental conditions
Cost and Infrastructure	Relatively low cost; widely accessible instrumentation	High cost and advanced instrumentation requirements
Clinical Translatability	Widely adopted in routine diagnostics and clinical research	Primarily applied in research and exploration clinical studies
Strengths	Operational simplicity, robustness, and clinical reliability	High-resolution, multidimensional characterization of innate immune status
Limitations	Limited ability to resolve subtle or complex immune perturbations	Barriers in standardization, scalability, and routine clinical implementation

### 3.3 Confounding factors affecting impact assessment

**Figure 7:**
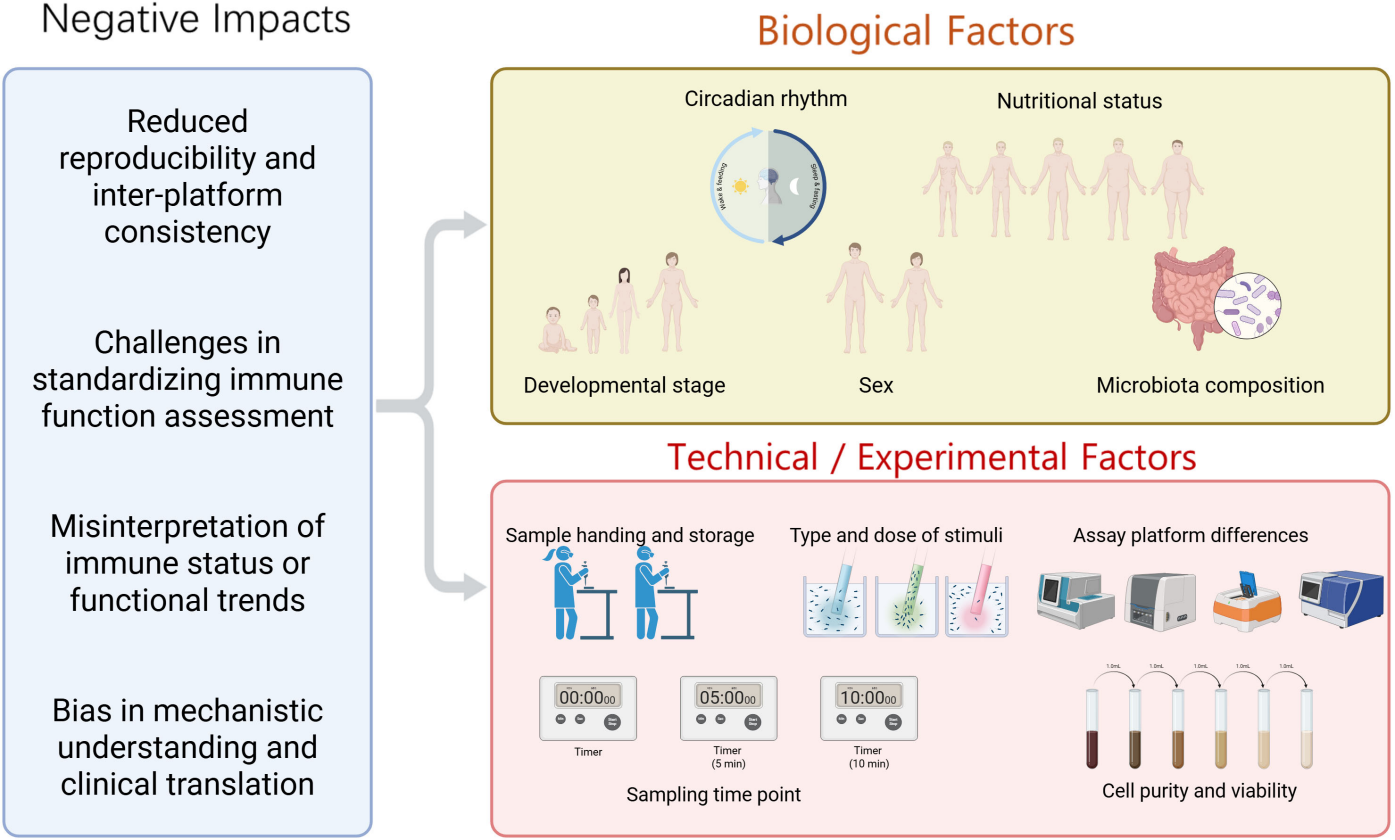
Biological variables (circadian rhythm, nutritional status, developmental stage, sex, microbiota composition) and technical and experimental factors (Sample handling and storage, sampling time points, type and dose of stimuli, assay platform differences, and cell purity and viability). These can substantially influence assay outcomes, thereby affecting reproducibility, cross-platform consistency, and interpretation of immune status and functional trends.

The standardization of systems, created to measure innate immune performance, is constantly assaulted by a range of confounding factors as presented in **Figure 7**. The stability, reproducibility, and comparability of test results are damaged by many different factors that lead to significant changes, usually resulting in substantial variability, which complicates the development of universally applicable evaluation standards, integration of data across experimental platforms, and the possibility of misinterpretation of immune functioning, which affects mechanistic understanding and translation to clinical applications (67, 68).

The difference in sample handling, quality of the reagents used, or the equipment may also lead to biases in the detection findings in other experimental or clinical contexts, which also propagates their scientific validity and applicability (69). The level of immune biomarkers when measuring the innate immunity status not only depends on the exposure to pathogens, but also on various external factors. These possible infective factors should be well taken into consideration when assessing immune functions, as shown in **Table 4**. To systematize and standardize testing of the innate immune functioning, a framework that is flexible and broadly adaptive to suitability to improve methodological rigor and interpretability of the results as well as allowing the passage between exploratory research and standardized clinical practice, it is necessary to have such a structure. Personalized and detailed approaches to immune surveillance, which combine several measurements of the biomarkers and specific clinical evaluations, are mandated to provide a clear under-standing of the condition of the immune system.

**Table 4:** External factors affecting assay outcomes.

External factors	Mechanisms affecting immune markers	References
Environmental Temperature	Cold stress increases levels of inflammatory factors (e.g., IL-1β, TNF-α, and IL-6) through activation of the sympathetic nervous system and release of adrenaline and other stress hormones. Chronic exposure may trigger a chronic inflammatory response.	(97)
Air Pollution and Chemicals	Prolonged exposure to air pollutants such as ozone suppresses the immune response, reduces phagocytosis by macrophages and increases cytokine secretion, leading to over-activation of the immune system and possibly triggering immune fatigue.	(98)
Nutritional State	Both under- and over-nutrition can significantly alter the intensity of the immune response. For example, chronic low-grade inflammation induced by a high-fat diet is strongly associated with cytokines (e.g., IL-6, TNF-α) secreted by adipose tissue. Immune cell function is diminished in undernutrition.	(99, 100)
Stress Response	Chronic psychological or physiological stress (e.g., high-intensity exercise, emotional stress) alters the immune response through activation of the sympathetic nervous system and secretion of stress hormones (e.g., cortisol). Cortisol suppresses immune function, leading to a decrease in the immune system’s resistance to pathogens.	(36, 70, 101–103)

## 4 Conclusions and future perspective

**Figure 8:**
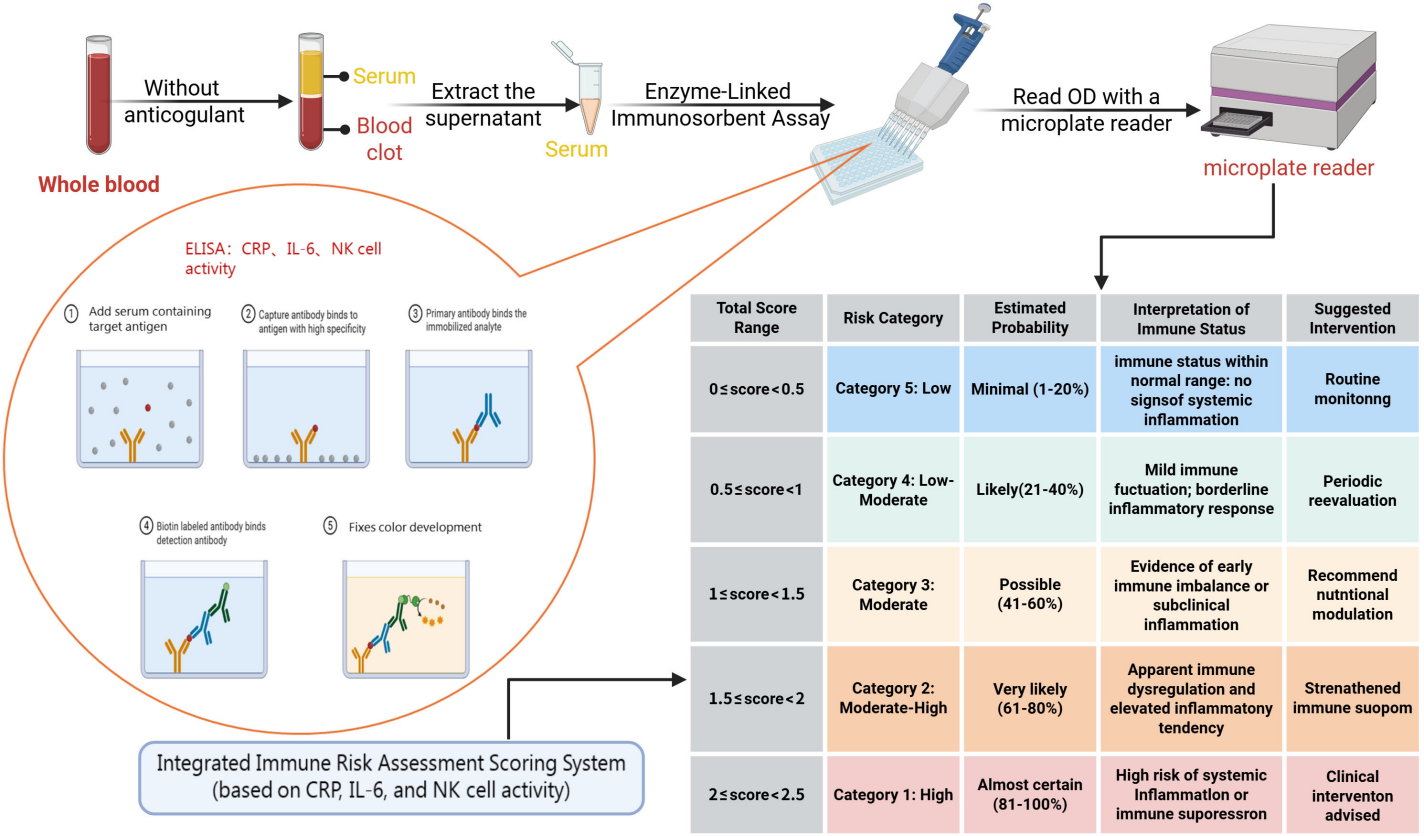
To measure the levels of CRP, IL-6, and NK cell activity in the blood, serum is extracted and centrifuged, after which the serum is used in ELISA to produce the corresponding readouts. The composite score is then obtained by calculated according to a pre-established formula and plotted into an immune intensity grading table where there are five levels of risk: Level 5 (0 ≤ score < 0.5) represents a low-risk condition where there is no inflammation of the system and no immediate follow-up; Level 4 (0.5 ≤ score < 1) is a low-to-moderate-level of risk, which may represent low-grade inflammatory responses, which should be followed using periodic reassessment; Level 3 (1 ≤ score < 1.5) represents moderate risk, suggesting early immune imbalance or subclinical inflammation, with nutritional interventions advised; Level 2 (1.5 ≤ score < 2) indicates elevated risk, manifesting as evident immune dysregulation and inflammatory tendency, signaling the need for enhanced immune support; Level 1 (2 ≤ score < 2.5) denotes high risk, reflecting systemic inflammation or potential immuno-suppression, necessitating clinical intervention.

A universal system of evaluating the functioning of the innate immune system has a big promise in both clinical disciplines. Nevertheless, despite decades of studying innate immunity, significant obstacles remain when attempting to standardize the evaluation of the immune system and monitor dynamic changes. Biomarkers can be introduced through modular integration to increase the analytical resolution and applicability across the environment, which can give the innate immune status assessment a powerful platform to model and develop algorithms. The dynamic variations of the immune system can be better demonstrated by multivariate correlation analysis and multidimensional analysis. Indicatively, research studies have found that the ‘high CRP/IL-6 + low NK cell activity’ pattern represents a characteristic immunological feature in critically ill patients. This pattern correlates laboratory markers with clinical severity (ICU admission) (63, 70). Given the limitations of the available dataset, detailed stratified analyses according to age and sex were not performed. Therefore, a simplified composite analytical framework was constructed and presented herein as an illustrative example. **Table 5** gives detailed scoring criteria, and the mechanism behind it is shown in **Figure 8**.

**Table 5:** Example of a simplified immune status assessment system comprising CRP, IL-6, and NK Cells.

Indicator name	Measurement methods	Risk scoring criteria	Percentage (%)	Instructions
CRP	ELISA	<5 mg/L = 0 score; 5–50 mg/L = 1 score; >50 mg/L = 2 score	30%	Reflects the level of systemic inflammation, with high values suggesting an increased risk of inflammation
IL-6	ELISA	<7 pg/mL = 0 score; 7–40 pg/mL = 1 score; >40 pg/mL = 2 score	40%	Reflects inflammation and immune activation, with higher levels suggesting an immune overreaction.
NK Cell Activity	ELISA	>80% (or normal reference lower limit) = 0 score; 50-80% = 1 score; <50% = 2 score	30%	Reflects innate immune cell killing function, low activity suggests immunosuppression

Composite score is determined in the following manner: Composite Score = (CRP Risk Score 50) + (IL-6 Risk Score 50) + (NK Activity Risk Score 50) = (CRP Risk Score 30%) (IL-6 Risk Score 40%) (NK Activity Risk Score 30%). CRP reflects systemic inflammatory burden as a stable downstream marker; markedly elevated levels (>50–100 mg/L) indicate persistent inflammation. A moderate weight was assigned due to its robustness, but limited specificity.IL-6 represents a key upstream driver of inflammatory amplification and immune regulation; levels >40 pg/mL are strongly associated with severe disease and immune suppression. Accordingly, the highest weight was assigned cell activity directly reflects innate immune effector function; substantially reduced activity (<50%) indicates immunodeficiency. Given its functional relevance and methodological complexity, a weight comparable to CRP was applied. The composite score has a scale of 0 to 2, where a high score would reflect a greater range of deviations of the innate immune status out of the healthy range, and this might be a greater risk of inflammatory imbalance or vice versa.

Reduction of inter-laboratory variability and improved reproducibility are based on standardization of the operational procedures and data workflows. However, standardized protocols need to be implemented to promote uniformity in the sample handling, experimental conditions, and data normalization and selection of reagents (71). The existing studies do not have a common model of response, and the norming of interpretation on the definition of a threshold and data normalization undermines the reproducibility and translational suitability. The vague measures of immunodeficiency and hyperactivity make it difficult to assess and give precise intervention. Subsequent analysis of innate immunity state must involve confound-er-correction schemes and powerful normalization algorithms, which will enhance the data comparability, which will eventually benefit standardized public health and personalized medicine. There is high heterogeneity among populations, age groups, and types of disease, whereas some major covariates, including the genetic background and nutrition, are usually not adequately addressed, which causes a substantial difference in the outcomes of assessment. TNF-α, IL-6, and IL-1β are the most common cytokines that are used as indicators of immune reaction activation during inflammatory reactions, but all the cytokines are extremely sensitive to external and patient-specific influences. This will require the formulation of integrated assessment systems that will consider personal variation (37). It is important to establish clinically relevant immune reference ranges throughout the human lifespan and establish standard, cross-platform comparable measurements. The refined population immune health reference ranges have to be determined by stratification in terms of age, sex, physiological status, and personal health. The standardization of innate immune testing needs to move beyond the days of using single-marker detection formats and move into the era of multidimensional analyses, in terms of bioinformatics networks. It involves combining massively scaled studies, statistical modeling, and machine learning in order to create dynamic, adaptive thresholds as opposed to using static cutoffs. A core version of an extended indicator framework is suggested: identify key immune molecules developed stably as core indicators and expand flexibly based on a particular research or clinical goal to include large-scale data and machine learning to set dynamic and adaptive evaluation thresholds instead of fixed and flexible standards. As one such example, within the context of inflame-aging, early signs of innate immune imbalance are key indicators of immune aging, and this is complementary to chronic low-grade inflammation, which can closely be linked with numerous age-related diseases. Assessment instruments may be systematic to enable the early detection of such states, which can be used to support accurate health response interventions in the aging population (72). Models that possess immune qualities similar to those of humans can be useful in human immunological studies, e.g., pigs. A stratified comparative immunology methodology may be considered in the promotion of standardized innate immunity testing to offset the discovery of universal immune principles, with consideration of specific peculiarities. Innate immune response is very dynamic and depends on stress, nutrition, exposure to microbes, and circadian cycles. The magnitude, duration, and recovery capacity of the natural immune system should be combined as assessment standards. The emergence of powerful tools in the creation of large-scale databases and tools of artificial intelligence models offers a potent force for building a generalized standardized system of assessment of innate immunity (73).

Proper and standardized assessment of innate immune status became the foundation of contemporary immunology and has extensive use in precision medicine, population health, and animal breeding. In healthcare for humans, it assists in testing the effectiveness of vaccines, chronic illnesses, and aging well. In addition to standard laboratory tests, vivo and ex vivo monitoring can become a new trend; non-invasive samples (e.g., saliva, nasal swabs) might provide real-time delivery and could be combined with wearable devices (74). Reduction of confounding factor interference is also a major technical issue. Since the innate immune system is highly complex, there are several confounding factors, advanced immunological and molecular methods, and the lack of integration of basic research and clinical practice; the priority in this case should be the goal to create a triad framework, including the development of technology, standardized protocols, and clinical implementation. Integrating quantification of cytokines and immune cell analysis is useful in the study of infectious diseases, where the dynamics of immune escape are noted, and ratio-based assessment models would be applicable to other autoimmune disease-related studies. The multidimensional assessment system must cover functional indicators on the levels of inflammation, cellular activity, metabolism, and epigenetic regulation. Mult omics mRNA data, behavioral parameters, clinical data, and wearable biosensors readings should be included to make the minimal invasive monitoring of the immune system in real-time and thus elucidate precision health management. In this way, the innate immune status assessment strategy is simple, instead of measuring individual biomarkers in a static way.

Future directions to address current limitations and guide development include:

1. Selection of innate immunity-related indicators, defining their health reference ranges and applicable conditions.
2. Strengthening cross-disciplinary technological collaboration, updating and developing novel detection and analysis methods.
3. Developing innate immune system monitoring models tailored to different populations.
4. Establishing AI-based multimodal data integration platforms for real-time, re-mote immune analysis.
